# Supplementary material for: Projection of the health and economic impacts of Chronic kidney disease in the Chilean population
Source: PLoS One. 2021 Sep 8;16(9):e0256680. doi: 10.1371/journal.pone.0256680 (PMC8425564; doi:10.1371/journal.pone.0256680)
Supplement: S1 Fig — Source: Kidney Disease: Improving Global Outcomes group. The numbers in the grid are the recommendations by KDIGO to the frequency of monitoring (in times per year). (PDF) [file pone.0256680.s001.pdf]

S1 Fig. Classification of CKD

				Persistent Albuminuria Categories Description and Range		
				A1	A2	A3
				Normal to mildly increased	Moderately increased	Severely increased
				ACR <30 mg/g	ACR of 30-300 mg/g	ACR >300 mg/g
GFR Categories (mL/min per 1.73 m_2) Description and Range	G1	Normal or high	≥90	1 if CKD	1	2
	G2	Mildly decreased	60-89	1 if CKD	1	2
	G3a	Mildly to moderately decreased	45-59	1	2	3
	G3b	Moderately to severely decreased	30-44	2	3	3
	G4	Severely decreased	15-29	3	3	4+
	G5	Kidney failure	<15	4+	4+	4+

Source: Kidney Disease: Improving Global Outcomes group. The numbers in the grid are the recommendations by KDIGO to the frequency of monitoring (in times per year).
