# Supplementary figures and images for: Projection of the health and economic impacts of Chronic kidney disease in the Chilean population
Source: PLoS One. 2021 Sep 8;16(9):e0256680. doi: 10.1371/journal.pone.0256680 (PMC8425564; doi:10.1371/journal.pone.0256680)

S2 Fig. Dynamic stock and flow model.

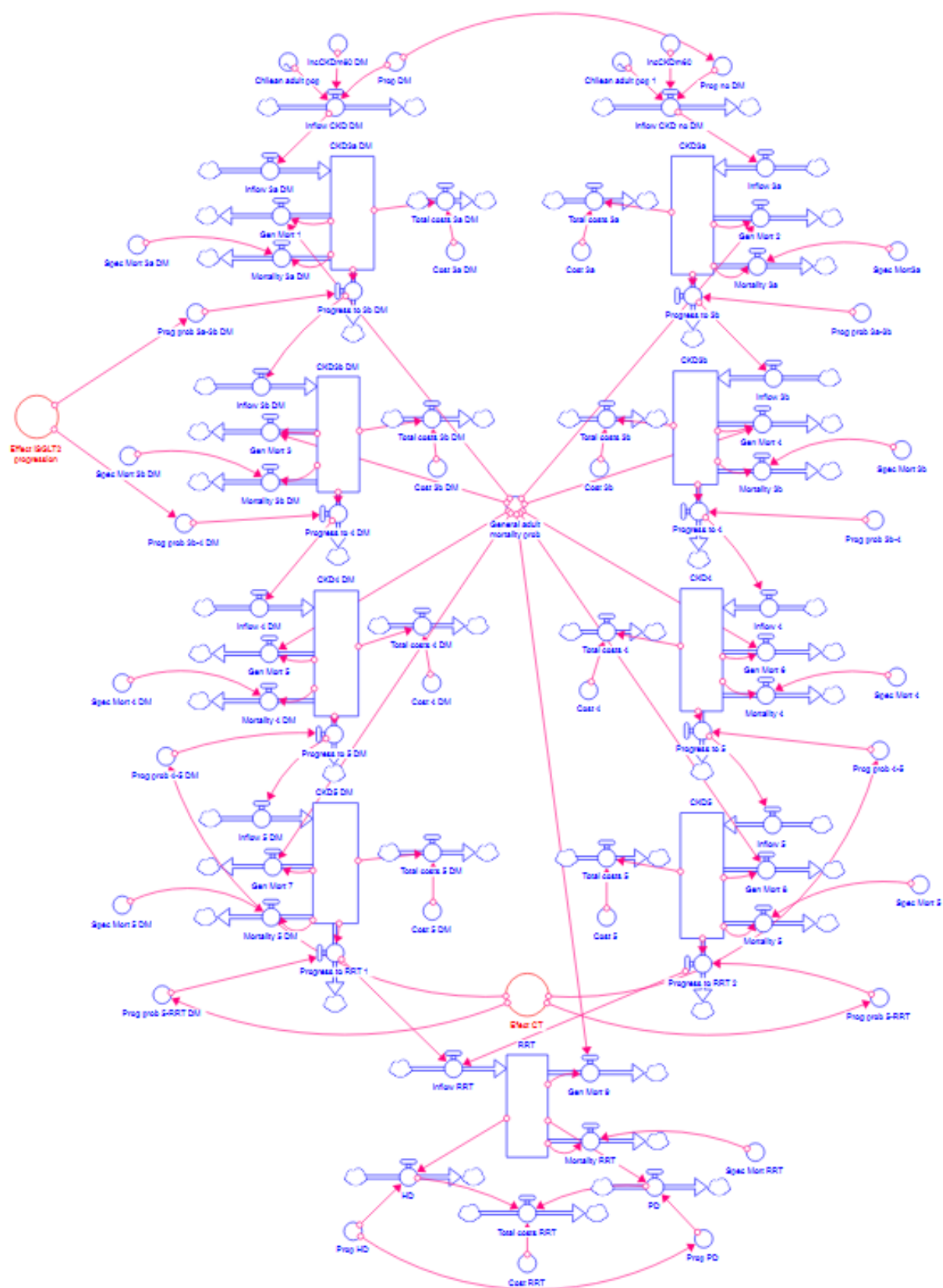

Dynamic Stock and flow model by Stella Professional V2.1.

Supplement: S2 Fig — Dynamic Stock and flow model by Stella Professional V2.1. (PDF) [file pone.0256680.s002.pdf]
