# Supplementary material for: Projection of the health and economic impacts of Chronic kidney disease in the Chilean population
Source: PLoS One. 2021 Sep 8;16(9):e0256680. doi: 10.1371/journal.pone.0256680 (PMC8425564; doi:10.1371/journal.pone.0256680)
Supplement: S3 Fig — The estimated glomerular filtration rate (eGFR) levels were calculated using the CKD-EPI equation based on the data of the two most recent Chilean national health surveys (ENS 2009–10 and 2016–17). The distribution was assessed for eGFR <60 ml/min/1.73 m2 with normal or increased albuminuria to estimate the proportion of individuals in each stage that would progress in one cycle to the next stage. (PDF) [file pone.0256680.s003.pdf]

S3 Fig. Distribution of eGFR in Chilean national health surveys (ENS 2009-10 and 2016-17).

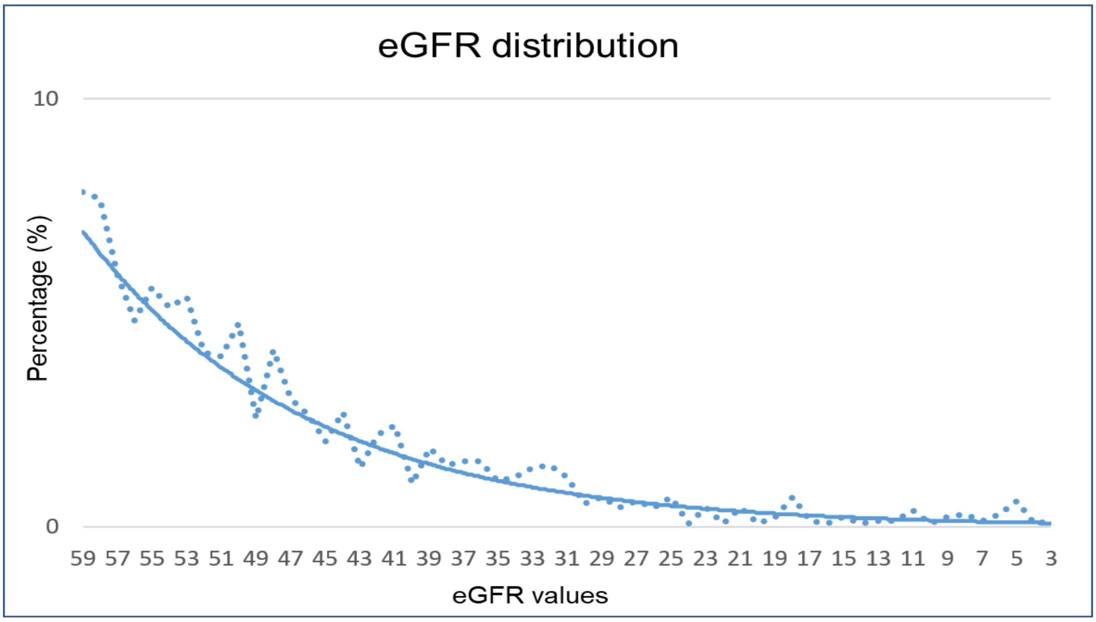

The estimated glomerular filtration rate (eGFR) levels were calculated using the CKD-EPI equation based on the data of the two most recent Chilean national health surveys (ENS 2009-10 and 2016-17). The distribution was assessed for eGFR <60 ml/min/1.73 m^2 with normal or increased albuminuria to estimate the proportion of individuals in each stage that would progress in one cycle to the next stage.
