# Supplementary material for: Projection of the health and economic impacts of Chronic kidney disease in the Chilean population
Source: PLoS One. 2021 Sep 8;16(9):e0256680. doi: 10.1371/journal.pone.0256680 (PMC8425564; doi:10.1371/journal.pone.0256680)
Supplement: S1 Table — Adapted from data extracted from the Chilean Individual Expected Cost Verification Study (EVC), the Chilean National Health Fund and experts’ opinion. a Annual use per patient. b Frequency of use considered as the percentage of patients that would use the specific treatment. c For simplification, we grouped all the laboratory tests considered for individuals in stages 3a and 3b. d Statins included: atorvastatin, lovastatin and pravastatin. e ACE inhibitors: Angiotensin-converting enzyme (ACE) inhibitors. Enalapril and captopril are included. f ARBs: Angiotensin receptor blockers (ARBs) or angiotensin II receptor antagonists. Losartan potassium is included. (PDF) [file pone.0256680.s004.pdf]

S1 Table. Treatment included for CKD stages 3a and 3b.

Treatment	Annual use[a]	Frequency of use[a]	Total Costs per treatment
Specialist consultation	1	100%	14.14
General physician consultation	3	100%	16.12
Nurse consultation	3	70%	3.02
Laboratory tests[c]	2	100%	8.57
Acetylsalicylic acid	365	80%	1.20
Statins[d]	365	40%	2.10
Furosemide	365	2%	0.04
Hydrochlorothiazide	365	40%	0.75
ACE inhibitors[e]	365	70%	0.79
ARBs[f]	365	30%	2.03
**Total costs stages 3a and 3b**			**48.76**

Adapted from data extracted from the Chilean Individual Expected Cost Verification Study (EVC), the Chilean National Health Fund and experts' opinion.

[a] Annual use per patient.

[b] Frequency of use considered as the percentage of patients that would use the specific treatment.

[c] For simplification, we grouped all the laboratory tests considered for individuals in stages 3a and 3b.

[d] Statins included: atorvastatin, lovastatin and pravastatin.

[e] ACE inhibitors: Angiotensin-converting enzyme (ACE) inhibitors. Enalapril and captopril are included.

[f] ARBs: Angiotensin receptor blockers (ARBs) or angiotensin II receptor antagonists. Losartan potassium is included.
