# Supplementary material for: Projection of the health and economic impacts of Chronic kidney disease in the Chilean population
Source: PLoS One. 2021 Sep 8;16(9):e0256680. doi: 10.1371/journal.pone.0256680 (PMC8425564; doi:10.1371/journal.pone.0256680)
Supplement: S2 Table — Adapted from data extracted from the Chilean Individual Expected Cost Verification Study (EVC), the Chilean National Health Fund and experts’ opinion. a Annual use per patient. b Frequency of use considered as the percentage of patients that would use the specific treatment. c For simplification, we grouped all the laboratory tests considered for individuals in stage 4. d ACE inhibitors: Angiotensin-converting enzyme (ACE) inhibitors. Enalapril and captopril are considered. e ARBs: Angiotensin receptor blockers (ARBs) or angiotensin II receptor antagonists. Losartan potassium is considered. f Intravenous iron is considered for 30% of patients with stage 4, one or two times a week. g Erythropoietin is considered for 30% of patients with stage 4 two times a week. (PDF) [file pone.0256680.s005.pdf]

S2 Table. Treatment for CKD stage 4.

Treatment	Annual use[a]	Frequency of use[b]	Total Costs per treatment
**Baseline treatment**			
Specialist consultation	3	100%	42.41
Nurse consultation	6	100%	8.64
Laboratory tests[c]	3	100%	12.86
ACE inhibitors[d]	365	70%	0.79
ARBs[e]	365	30%	2.03
**Considered for the pre-dialysis treatment**			
Psychologist consultation	1	100%	3.52
Physical therapy	3	100%	4.74
Calcium	365	40%	109.09
Vitamin D	12	50%	3.70
Intravenous iron[f]	5	30%	23.16
Erythropoietin[g]	104	30%	150.02
**Total costs stage 4**			**66.73**
**Total costs stage 4 with pre-dialysis treatment**			**360.97**

Adapted from data extracted from the Chilean Individual Expected Cost Verification Study (EVC), the Chilean National Health Fund and experts' opinion.

[a] Annual use per patient.

[b] Frequency of use considered as the percentage of patients that would use the specific treatment.

[c] For simplification, we grouped all the laboratory tests considered for individuals in stage 4.

[d] ACE inhibitors: Angiotensin-converting enzyme (ACE) inhibitors. Enalapril and captopril are considered.

[e] ARBs: Angiotensin receptor blockers (ARBs) or angiotensin II receptor antagonists. Losartan potassium is considered.

[f] Intravenous iron is considered for 30% of patients with stage 4, one or two times a week.

[g] Erythropoietin is considered for 30% of patients with stage 4 two times a week.
