# Supplementary material for: Projection of the health and economic impacts of Chronic kidney disease in the Chilean population
Source: PLoS One. 2021 Sep 8;16(9):e0256680. doi: 10.1371/journal.pone.0256680 (PMC8425564; doi:10.1371/journal.pone.0256680)
Supplement: S3 Table — Adapted from data extracted from the Chilean Individual Expected Cost Verification Study (EVC), the Chilean National Health Fund and experts’ opinion. a Annual use per patient. b Frequency of use considered as the percentage of patients that would use the specific treatment. c For simplification, we grouped all the laboratory tests considered for individuals in stage 5. d ACE inhibitors: Angiotensin-converting enzyme (ACE) inhibitors. Enalapril and captopril are considered. e ARBs: Angiotensin receptor blockers (ARBs) or angiotensin II receptor antagonists. Losartan potassium is considered. f Intravenous iron is considered for 50% of patients with stage 5, one time per month. g Erythropoietin is considered for 50% of patients with stage 5, two times a week. (PDF) [file pone.0256680.s006.pdf]

S3 Table. Treatment for CKD stage 5.

Treatment	Annual use[a]	Frequency of use[b]	Total Costs per treatment
**Baseline treatment**			
Specialist consultation	4	100%	56.55
Nurse consultation	6	100%	8.64
Laboratory tests[c]	6	100%	25.72
ACE inhibitors[d]	365	70%	0.79
ARBs[e]	365	30%	2.03
Oral furosemide	730	20%	682.04
Oral bicarbonate	1095	20%	214.35
**Considered for the pre-dialysis treatment**			
Psychologist consultation	2	100%	7.04
Physical therapy	3	100%	4.74
Vitamin D	12	40%	2.96
Calcium	365	100%	272.73
Intravenous iron[f]	12	50%	92.65
Erythropoietin[g]	104	50%	250.04
Ketoanalogue	420	15%	17.18
**Total costs stage 5**			**990.12**
**Total costs stage 5 with pre-dialysis treatment**			**1637.46**

Adapted from data extracted from the Chilean Individual Expected Cost Verification Study (EVC), the Chilean National Health Fund and experts' opinion.

[a] Annual use per patient.

[b] Frequency of use considered as the percentage of patients that would use the specific treatment.

[c] For simplification, we grouped all the laboratory tests considered for individuals in stage 5.

[d] ACE inhibitors: Angiotensin-converting enzyme (ACE) inhibitors. Enalapril and captopril are considered.

[e] ARBs: Angiotensin receptor blockers (ARBs) or angiotensin II receptor antagonists. Losartan potassium is considered.

[f] Intravenous iron is considered for 50% of patients with stage 5, one time per month.

[g] Erythropoietin is considered for 50% of patients with stage 5, two times a week.
