# Supplementary material for: Projection of the health and economic impacts of Chronic kidney disease in the Chilean population
Source: PLoS One. 2021 Sep 8;16(9):e0256680. doi: 10.1371/journal.pone.0256680 (PMC8425564; doi:10.1371/journal.pone.0256680)
Supplement: S4 Table — Adapted from data extracted from the Chilean Individual Expected Cost Verification Study (EVC), the Chilean National Health Fund and experts’ opinion. HD: Haemodialysis. PD: peritoneodialysis. a Annual use per patient based on the Expected Cost Verification Study in 2019. b Frequency of use considered as the percentage of patients that would use the specific treatment. c For simplification, we included all the types of vascular access covered by Chilean healthcare system in this section. The election of it will depend on the need of the patient and decided by the specialist. d For simplification, we grouped all the laboratory tests considered for individuals in stage 5. e Intravenous iron is considered for all patients undergoing dialysis, around 3 times per month. f Erythropoietin is considered for all patients undergoing dialysis, two times a week for 9 months per year. (PDF) [file pone.0256680.s007.pdf]

S4 Table. Treatment included for ESKD.

Treatment	Annual use[a]	Frequency of use[b]	Total Costs per treatment
HD vascular access installation procedure[c]	1	50%	506.24
PD catheter installation procedure	1	2%	10.65
Dysfunctional and/or occluded vascular access repair	1	3.4%	46.10
Specialist consultation	3	100%	42.41
Nurse consultation	3	100%	4.32
Laboratory tests[d]	4	100%	17.15
Monthly HD	12	95%	9969.76
Monthly PD	12	5%	607.50
Intravenous iron[e]	32	100%	494.15
Erythropoietin[f]	72	100%	346.21
**Total costs ESKD**			**12044.48**

Adapted from data extracted from the Chilean Individual Expected Cost Verification Study (EVC), the Chilean National Health Fund and experts' opinion. HD: Haemodialysis. PD: peritoneodialysis.

[a] Annual use per patient based on the Expected Cost Verification Study in 2019.

[b] Frequency of use considered as the percentage of patients that would use the specific treatment.

[c] For simplification, we included all the types of vascular access covered by Chilean healthcare system in this section. The election of it will depend on the need of the patient and decided by the specialist.

[d] For simplification, we grouped all the laboratory tests considered for individuals in stage 5.

[e] Intravenous iron is considered for all patients undergoing dialysis, around 3 times per month.

[f] Erythropoietin is considered for all patients undergoing dialysis, two times a week for 9 months per year.
