# Supplementary material for: Projection of the health and economic impacts of Chronic kidney disease in the Chilean population
Source: PLoS One. 2021 Sep 8;16(9):e0256680. doi: 10.1371/journal.pone.0256680 (PMC8425564; doi:10.1371/journal.pone.0256680)
Supplement: S5 Table — Adapted from data extracted from the Chilean Individual Expected Cost Verification Study (EVC), the Chilean National Health Fund and experts’ opinion. DM: Diabetes Mellitus. a These costs consider only the treatment for DM that is not covered by the treatment of CKD (e.g. physicians consultations were not considered in the costing as we assumed the ones used for each CKD stage). b Annual use per patient based on the Expected Cost Verification Study in 2019. c Frequency of use considered as the percentage of patients that would use the specific treatment. (PDF) [file pone.0256680.s008.pdf]

S5 Table: Costs included for the treatment of DM[a]

Treatment	Annual use[b]	Frequency of use[c]	Total Costs per treatment
Specialist consultation	1	100%	14.14
Nutritional consultation	3	80%	3.46
Glycated haemoglobin exam	3	100%	12.96
Isophane insulin (NPH)	18	15%	5.24
Syringes	365	15%	4.68
Blood glucose monitor	365	15%	3.83
Metformin	730	70%	5.26
Sulfonylureas	1095	40%	1.80
**Total costs DM**			**51.37**

Adapted from data extracted from the Chilean Individual Expected Cost Verification Study (EVC), the Chilean National Health Fund and experts' opinion.

DM: Diabetes Mellitus

[a] These costs consider only the treatment for DM that is not covered by the treatment of CKD (e.g. physicians consultations were not considered in the costing as we assumed the ones used for each CKD stage)

[b] Annual use per patient based on the Expected Cost Verification Study in 2019.

[c] Frequency of use considered as the percentage of patients that would use the specific treatment.
